# Supplementary material for: Identification of photoperiod-induced specific miRNAs in the adrenal glands of Sunite sheep (Ovis aries)
Source: Front Vet Sci. 2022 Jul 22;9:888207. doi: 10.3389/fvets.2022.888207 (PMC9354845; doi:10.3389/fvets.2022.888207)
Supplement: Supplementary file 1 [file Data_Sheet_1.ZIP › Supplementary file/miRDeep2 file.zip!/miRDeep2 file/Novel_Structure/SP42A1/Novel_432.pdf]

Provisional ID	:	NC_019470.2_16759
Score total	:	2.4
Score for star read(s)	:	-1.3
Score for read counts	:	0
Score for mfe	:	2.1
Score for randfold	:	1.6
Score for cons. seed	:	
Total read count	:	24
Mature read count	:	23
Loop read count	:	0
Star read count	:	1

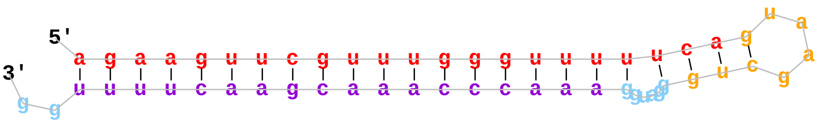

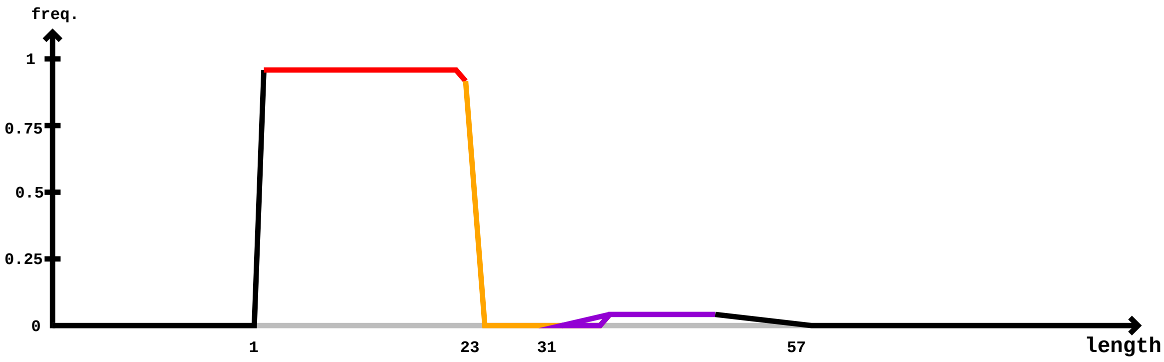


```
                                         Mature                                 Star
5'- aagauuuaucagaauggcccagaaguucguuuggguuuuucaguaagcugggauggaaacccaaacgaacuuuugggccaacccaauacauuuccaaauaggaaaaggagu   -3'   obs
    aagauuuaucagaauggcccagaaguucguuuggguuuuucaguaagcugggauggaaacccaaacgaacuuuugggccaacccaauacauuuccaaauaggaaaaggagu         exp
    ..............(((((((((((((((((((((((((((((....))))....))))))))))))))))))))))))).........(.(((((.....))))).)....   reads   mm   sample
    ....................agaaguucguuuggguuuuuc......................................................................   1       0    seq
    ....................aAaaguucguuuggguuuuuca.....................................................................   22      1    seq
    ........................................................aaacccaaacgaacuuuu.....................................   1       0    seq
```
